# Spontaneous intramural jejunal haematoma: a case report

**DOI:** 10.1186/1757-1626-1-389

**Published:** 2008-12-12

**Authors:** Rashmi P Birla, Kamal K Mahawar, Elena YW Saw, Mohamed A Tabaqchali, Philip Woolfall

**Affiliations:** 1 Department of General Surgery, University Hospital of North Tees, Hardwick Road, Stockton-on-Tees, Cleveland, UK; 2 Radiology, University Hospital of North Tees, Hardwick Road, Stockton-on-Tees, Cleveland, UK

## Abstract

**Background:**

Spontaneous intramural intestinal haematoma is a rare complication of anticoagulation therapy. A misdiagnosis may lead to an unnecessary and even hazardous surgical intervention.

**Case presentation:**

An 85 year old lady presented to us with acute abdomen. She was on Warfarin and was found to have grossly deranged clotting parameters. Computed Tomography Scan revealed a long loop of markedly thick-walled proximal jejunum. A diagnosis of spontaneous intramural jejunal haematoma was made.

**Conclusion:**

She was successfully treated with conservative management with Vitamin K and blood products.

## Background

Spontaneous intramural bowel wall haematoma is an often forgotten, rare clinical entity which may pose considerable diagnostic dilemma. It is important to correctly diagnose this condition in order to avoid unnecessary surgical intervention. We present here an interesting case and a brief review of the available literature.

## Case report

An 85-year old lady presented as an emergency with sudden onset of severe left sided abdominal pain and vomiting for two days. This was associated with constipation for 3 days. She had a past medical history of atrial fibrillation, angina, and pulmonary embolism for which she was anticoagulated with warfarin. There was no history of trauma.

On examination she was haemodynamically stable. Her abdomen was generally tender, maximal tenderness being in the left hypochondrium and left lumbar region, associated with some guarding and rigidity. Bowel sounds were present and the rectal examination was unremarkable.

**Figure 1:**
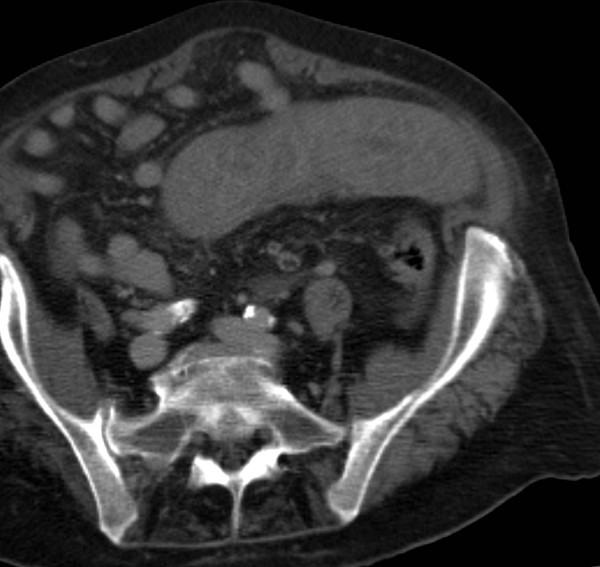
**Axial CT scan**. The grossly thickened loop of jejunum on the left side of the abdomen is typical of intramural haematoma.

**Figure 2:**
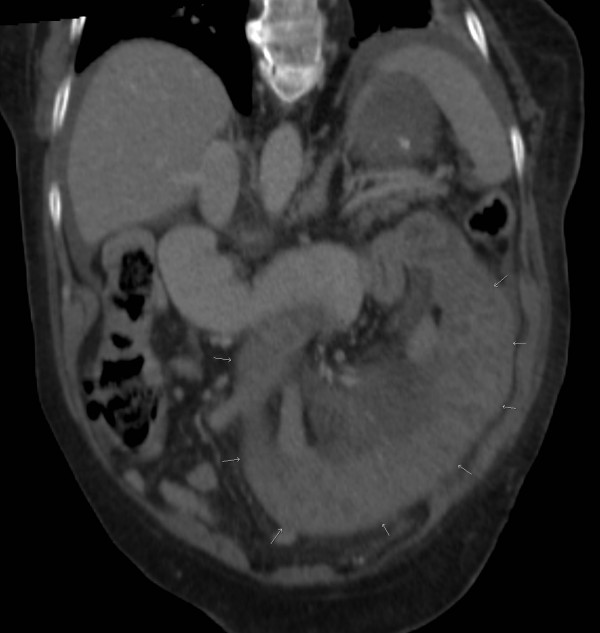
**Reformatted CT scan of intramural haematoma of jejunum**. This image reformatted in the coronal plane shows the full extent of the abnormal bowel (arrows). A relatively long segment of abnormal bowel (around 20 cm) is typical. There is free fluid around the spleen in the left upper quadrant.

The blood tests revealed normal haemoglobin, marginally raised white cell count (12.5) and C- reactive protein (25.3), and a grossly deranged International Normalised Ratio (INR) of 8.9. Her renal and liver functions function tests were within normal limits. An erect Chest X-ray and supine abdominal films were both unremarkable. Computed Tomography (CT) Scan showed a long abnormal loop of thick walled jejunum with a small amount of free fluid in the intraperitoneal cavity (Figure 1 and 2).

A diagnosis of spontaneous intramural haematoma of the jejunum was made. She was treated conservatively with nil by mouth, intravenous fluids, and nasogastric suction. Fresh frozen plasma and vitamin K were given to normalise her clotting parameters. Her symptoms subsided gradually over the next couple of days and she was gradually started on fluid and diet. The clotting parameters normalized and she was discharged home on normal diet six days later.

## Discussion

Spontaneous intestinal intramural haematoma is an uncommon complication of anticoagulation. Before the advent of anticoagulant therapy, most cases of intramural haematoma were related to trauma [1]. In 1965 Walter Goldfarb published a series of eleven patients with intestinal haemorrhage related to oral anticoagulant therapy [2]. Warfarin toxicity still remains the dominant cause accounting for the vast majority of patients [3]. Other risk factors include haemophilia, idiopathic thrombocytopenic purpura, leukemia, lymphoma, myeloma, chemotherapy, vasculitis, pancreatitis, and pancreatic cancer [3]. Patients on conventional heparin rarely suffer from this condition, presumably because they are in-patient and undergo stricter monitoring of clotting parameters. We did however find one report in literature of small bowel haematoma in a child on low molecular weight heparin for deep venous thrombosis [4].

Incidence of spontaneous intramural haematoma is reported to be 1 per 2500 anticoagulated patients [5]. The mean age at presentation in one recent series of 13 patients was 64 years; 15% of the patients in this series had multiple haematoma [3]. Though also seen in the ileum, duodenum and colon, the jejunum remains the commonest site (69%) [5-8]. Whether this is due to the relative fixity of the jejunum to the ligament of Treitz or some other characteristic of the jejunum is not entirely clear.

The presentation can vary from mild, vague abdominal pain to intestinal obstruction and an acute abdomen [3]. The classic clinical triad of abdominal pain, small bowel obstruction and multiple haemorrhagic symptoms may not be seen in all the patients [5]. Some patients have other haemorrhagic manifestations e.g., major upper GI haemorrhage [3,9]; but Beamish et al noted that this is not a constant finding [10]. Small bowel obstruction, often incomplete, is the commonest mode of presentation [2]. The diagnosis is often not suspected initially and established only on imaging or laparotomy.

If suspected pre-operatively, diagnosis usually requires CT scanning for confirmation. An Ultrasound (US) scan [8] or an upper GI contrast series [3] may sometimes prove useful. In a study by Catalano et al [8], characteristic US findings were double or multilayered thickening of the bowel wall (usually with a thick and hyperechoic inner layer and a thin and hypoechoic outer layer), undulated mucous membrane, narrowed lumen, decreased peristalsis with fixity of the images, and fluid between the loops. An upper gastrointestinal contrast study should be unnecessary, but if performed, may show a stacked-coin appearance representing a thickening of folds with sharp demarcation and crowding of valvulae conniventes, a picket fence appearance showing spike like projections of barium between adjacent thickened mucosal folds, abrupt proximal and distal transition points, and luminal narrowing of a rigid and non distensible segments of intestine [3].

CT Scan is the investigation of choice. It has been suggested that an initial non-contrast scan should be performed to help demonstrate high attenuation haemorrhage in the bowel wall [6]. The finding of a long segment of bowel wall thickening in the jejunum in an anticoagulated patient strongly suggests the diagnosis. Ascites, as seen in our patient, is a common finding.

Historically, most cases of spontaneous intestinal haematoma have been diagnosed at laparotomy [2,3]. If correctly diagnosed pre-operatively, conservative management with restoration of coagulation parameters leads to a satisfactory recovery in most cases. Surgery should be reserved for patients exhibiting signs of frank generalized peritonitis due to bowel necrosis [4], in cases where conservative treatment fails to resolve symptoms, or where diagnosis cannot be established with confidence. A follow up CT scan may be carried out at a 2–3 month interval to ensure complete resolution of haematoma. Persistent lesions should be subjected to further investigation. Studies have shown that resolution starts as early as one week after the insult and can take up to two months for complete recovery [3].

## Conclusion

Spontaneous small bowel haematoma is a rare clinical entity. It should be considered in any patient on long term anticoagulation therapy presenting with an acute abdomen. The jejunum is the commonest site. A high index of suspicion is required to manage these patients appropriately and avoid unnecessary laparotomy.

## Abbreviations

INR: International Normalised Ratio; US: Ultrasound; CT: Computed Tomography.

## Consent

Written informed consent was obtained from the patient for publication of this case report and accompanying images. A copy of the written consent is available for review by the Editor-in-Chief of this journal.

## Competing interests

The authors declare that they have no competing interests.

## Authors' contributions

ES collected the data on the patient and contributed to the literature search in the manuscript. RB helped with writing the manuscript. KM conceived the idea and helped with the draft of the case report. AR was involved in the care of patient. MT was the consultant-in-charge of patient and supervised the effort. PW interpreted the radiological scans, contributed the images and helped to write the manuscript. All authors read and approved the final manuscript.
